# A Comparison of Patients Undergoing On- vs. Off-Pump Coronary Artery Bypass Surgery Managed with a Fast-Track Protocol †

**DOI:** 10.3390/jcm10194470

**Published:** 2021-09-28

**Authors:** Henrike Grützner, Anna Flo Forner, Massimiliano Meineri, Aniruddha Janai, Jörg Ender, Waseem Zakaria Aziz Zakhary

**Affiliations:** 1 Section for Pediatrics and Youth Medicine, Public Health Department, Leipzig City Government, Friedrich-Ebert-Straße 19 a, 04109 Leipzig, Germany; henrike.gruetzner@leipzig.de; 2 Department of Anesthesiology and Intensive Care Medicine, Heart Center Leipzig, Strümpellstraße 39, 04289 Leipzig, Germany; anna.floforner@helios-gesundheit.de (A.F.F.); Massimiliano.Meineri@helios-gesundheit.de (M.M.); aniruddha.janai@helios-gesundheit.de (A.J.); Joerg.Ender@helios-gesundheit.de (J.E.)

**Keywords:** fast-track cardiac anesthesia, recovery area, on-pump coronary artery bypass surgery, off-pump coronary artery bypass surgery, ventilation time

## Abstract

The purpose of this study was to compare patients who underwent on- vs. off-pump coronary artery bypass surgery managed with a fast-track protocol. Between September 2012 and December 2018, *n* = 3505 coronary artery bypass surgeries were managed with a fast-track protocol in our specialized post-anesthesia care unit. Propensity score matching was applied and resulted in two equal groups of *n* = 926. There was no significant difference in ventilation time (on-pump 75 (55–120) min vs. off-pump 80 (55–120) min, *p* = 0.973). We found no statistically significant difference in primary fast-track failure in on-pump (8.2% (76)) vs. off-pump (6% (56)) groups (*p* = 0.702). The secondary fast-track failure rate was comparable (on-pump 12.9% (110) vs. off-pump 12.3% (107), *p* = 0.702). There were no significant differences between groups in regard to the post-anesthesia care unit, the intermediate care unit, and the hospital length of stay. Postoperative outcome and complications were also comparable, except for a statistically significant difference in PACU postoperative blood loss in on-pump (234 mL) vs. off-pump (323 mL, *p* < 0.0001) and red blood cell transfusion (11%) and (5%, *p* < 0.001), respectively. Our results suggest that on- and off-pump coronary artery bypass surgery in fast-track settings are comparable in terms of ventilation time, fast-track failure rate, and postoperative complications rate.

## 1. Introduction

Coronary artery bypass grafting (CABG) can be performed with the use of cardiopulmonary bypass (on-pump-CABG) or without (off-pump CABG). Studies comparing on- to off-pump CABG have consistently reported longer postoperative mechanical ventilation time and longer ICU- and hospital length of stay in the on-pump group [1,2,3], possibly explained by aortic manipulation and cardiopulmonary bypass (CPB)-triggered pro-inflammatory response which increased the risk of myocardial damage, adverse neurologic events and renal injury [4].

Fast-track cardiac anesthesia aims to reduce ventilation time, intensive care unit (ICU)- and consequently hospital-length of stay to optimize resource utilization [5]. Fast tracking can be performed in the ICU or in a specialized post-anesthesia care unit (PACU), thus completely avoiding ICU admission [6]. Fast-track management through a PACU proved to be a safe option for cardiac surgical patients at low-to-moderate risk and leads to reduced ventilation time as well as reduced ICU length of stay [7,8]. Tight intraoperative temperature management, optimal surgical hemostasis and hemodynamic stability are the prerequisites for successful fast-tracking [9]. Furthermore, a fast-track protocol has been shown to be safe [10,11], improve outcome [12], and reduce costs [5,13], after both on- or off-pump CABG. However, only one study to date has compared these two surgical approaches for myocardial revascularization using the same protocol [14].

The aim of this study was to compare the postoperative course of patients undergoing on- vs. off-pump CABG managed through a specialized PACU and the same fast-track protocol [15]. Primary endpoints were mechanical ventilation time (from arrival in PACU to tracheal extubation) and primary fast-track failure (unplanned transfer from PACU to ICU or the operating room). Secondary endpoints included PACU, intermediate care unit (IMC) and in hospital length of stay, re-intubation rate, and secondary fast-track failure (transfer from IMC or ward to the ICU or IMC, respectively) and postoperative outcomes measurements (e.g., blood transfusion, major adverse cardiovascular events, mortality).

## 2. Materials and Methods

This retrospective study was approved by the local ethics committee (approval number 178/19-ek from 30 April 2019) and performed in a single heart center. Individual patient consent was waived, given the retrospective observational nature of the study. Inclusion criteria were on- or off-pump CABG and fast-track management through a specialized PACU between September 2012 and December 2018. Patients who were intraoperatively converted from off-pump to on-pump surgery were excluded from the study. Patients operated on between February and July 2017 were excluded because of a temporary change in a fast-track protocol due to a shortage of remifentanil.

### 2.1. Anesthesia Management

Dipotassium clorazepate 10–20 mg was orally administered as premedication the evening before the operation when deemed necessary. The induction of general anesthesia was achieved using propofol 1–2 mg/kg, fentanyl 200 µg and a single dose of rocuronium (0.5–0.6 mg/kg) or atracurium (0.3–0.6 mg/kg) I.V. for neuromuscular blockade. After endotracheal intubation, a three-lumen central venous catheter and 8.5 F introducer sheath were inserted in the right internal jugular vein under ultrasound guidance; body temperature was monitored in the bladder though a urinary catheter and using a nasopharyngeal temperature probe. Transesophageal echocardiography was performed only when indicated (i.e., reduced ejection fraction or known pulmonary hypertension). Finally, pulse contour analysis (Vigileo^®^, Edwards Lifesciences, California, CA, USA) was used for all off-pump cases. Anesthesia was maintained by a continuous infusion of remifentanil 0.2–0.3 µg/kg/min I.V. and sevoflurane at a minimum alveolar concentration of 0.8–1.1%. During CPB, patients received a continuous infusion of propofol 3 mg/kg/h I.V. For all off-pump cases, an external forced-air warming system (3M™ Bair Hugger™ Full Access Underbody Blanket 63500, Minnesota, MN, USA) was used from before induction until it was transferred to PACU and set to keep the core temperature ≥36 °C. The same system was used in on-pump CABG cases and turned on at the time of re-warming during CPB. It was finally utilized in the PACU if the core temperature was <36 °C on admission. All infusions and blood products were warmed though the LEVEL 1 HOTLINE^®^ Blood and Fluid Warmer (Smiths Medical, Minnesota, MN, USA).

Red blood cells’ transfusion was triggered by a hematocrit less than 20% during CPB, or less than 25% after weaning from CPB and during off-pump surgery.

### 2.2. Surgical Management

The decision to select on- or off-pump procedure as well as the type and number of grafts was based on the patient’s condition (e.g., porcelain aorta or mobile plaques) and the surgeon’s preference.

Intraoperatively, all patients received unfractionated heparin as anticoagulant and its effect was monitored using activated clotting time (ACT). For on-pump CABG, patients received 300–500 IU/kg heparin to reach an ACT above 480 s, while off-pump CABG patients received 150–250 IU/kg heparin to reach an ACT above 300 s.

CPB management protocol were the same for all on-pump patients. When needed, antegrade blood or crystalloid-based Bretschneider cardioplegia solution (Custodiol^®^ Dr. Franz Köhler Chemie GmbH, Bensheim, Germany) was used according to the surgeon`s preference. Normothermia (>34 °C) or mild hypothermia (32–34 °C) was maintained in most of cases during CPB.

### 2.3. Management in PACU

At the end of the surgery, when the fast-track criteria were met and agreed upon by both the surgeon and the anesthesiologist, the patient was transferred to PACU. Fast-track criteria were defined as hemodynamic stability, a core temperature of ≥36 °C, minimal inotropic support (continuous infusion of <0.1 mg/kg/min of norepinephrine and/or <0.05 mg/kg/min of epinephrine or <4 µg/kg/min of dobutamine) and satisfactory hemostasis. The PACU was open from Monday to Friday 10:00 a.m.–10:30 p.m. with one anesthesiologist every four patients and one nurse every three patients.

Upon arrival at the PACU, patients received metamizole 1 g I.V. and piritramide 0.1 mg/kg I.V. for postoperative analgesia. Extra boluses of piritramide 0.02–0.03 mg/kg I.V. were given as needed to achieve a pain numeric rating scale inferior to 4.

Patients were extubated when they were fully conscious and hemodynamically stable. Afterwards, they were monitored for a minimum of two hours and then transferred to the IMC when fulfilling the criteria determined by the fast-track protocol [15].

**Figure 1:**
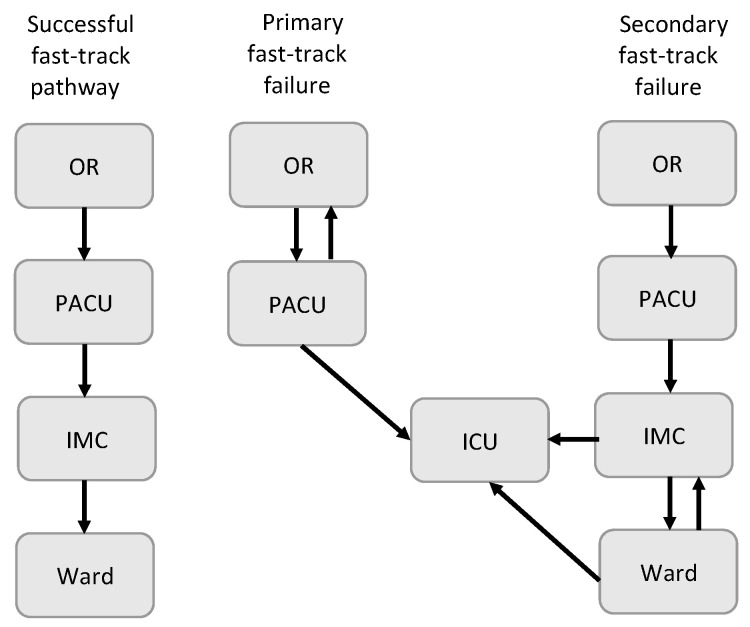
Types of fast-track failure. OR = operating room; PACU = postanesthetic care unit, IMC = intermediate care unit, ICU = intensive care unit.

Primary fast-track failure was defined as an unplanned transfer from the recovery unit to the ICU or back to the operating room. Transfers from the IMC or ward back to the ICU or IMC, respectively, were considered as secondary fast-track failure (Figure 1).

### 2.4. Data Collection and Analysis

The primary anesthesiologist scanned the anesthesia chart and the PACU observation chart using the machine-readable patient’s chart Medlinq^®^ software (Medlinq Softwaresysteme GmbH, Hamburg, Germany). Manual corrections were allowed before final charts’ saving in case of unreadable handwriting or inconsistencies. The author H.G. collected all data retrospectively from the clinical information system iMedOne^®^ (Deutsche Telekom Healthcare and Security Solutions GmbH, Bonn Germany), clinical information system Clemens^®^ (Teratec GmbH, Münster, Germany), and the patient’s chart Medlinq^®^. SPSS (SPSS^®^ Statistics 25.0; Chicago, IL, USA) and StatsDirect (StatsDirect^®^ version 3.0, StatsDirect Ltd., Cheshire, UK) were used for data description and analysis.

To guarantee two comparable groups and to minimize selection bias on the primary endpoint, we used propensity score matching. To build the logistic regression model, several variables, known to affect postoperative ventilation time and fast-track failure, were included. Specifically: age, gender, body mass index, American Society of Anesthesiologists physical status classification, logistic EuroSCORE, preoperative left ventricular ejection fraction, New York Heart Association Functional Classification, comorbidities, and duration of surgery. We used one-to-one matching and paired each subject to the closest propensity score subject from the other group. Based on the pre-matching range of baseline variable differences, the maximum caliper width for pair-matching was defined as 0.125 of the pooled logit score standard deviation.

Continuous variables were assessed for the normal distribution using the Shapiro–Wilk’s test. The data are expressed as the mean (standard deviation) and compared using Student’s t-test when normally distributed; otherwise, the results are expressed as median (interquartile range). Mann–Whitney-U-test was used for comparisons. Categorical data were expressed as numbers (proportion) and compared using the X^2^-test or Fisher’s exact test where appropriate. A *p*-value < 0.05 was considered statistically significant.

## 3. Results

**Figure 2:**
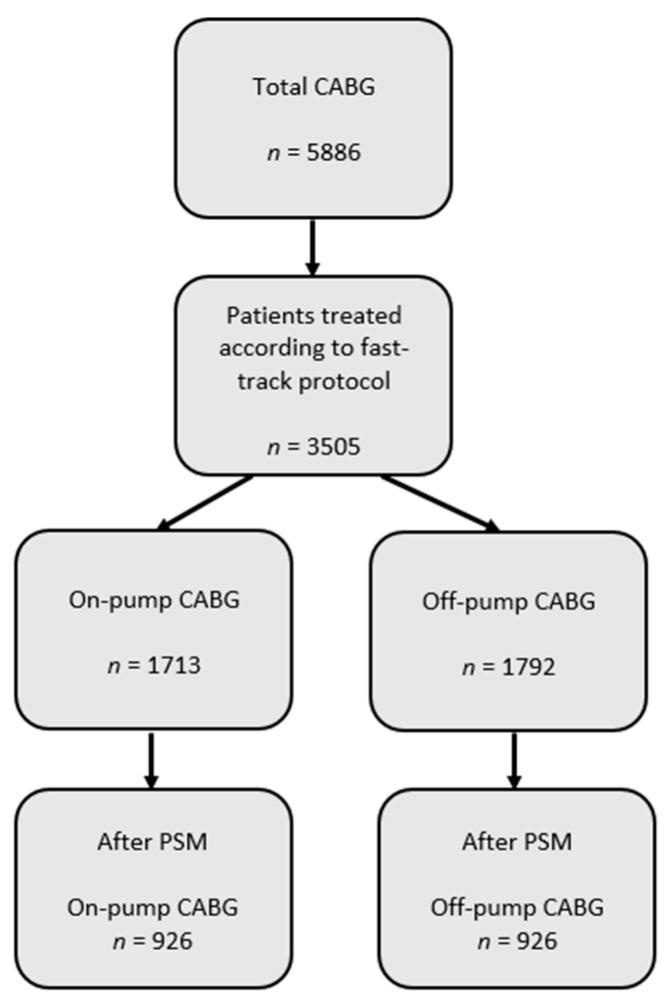
Patients flowchart. CABG = coronary artery bypass grafting; PSM = propensity score matching.

Between 2012 and 2018, 5886 coronary bypass operations were performed at our institution, 3505 of which were managed with a fast-track protocol. Propensity score matching yielded two equal groups *n* = 926, thus excluding overall 1653 patients (Figure 2).

The patient’s baseline characteristics and operative data were comparable and shown in Table 1.

**Figure 3:**
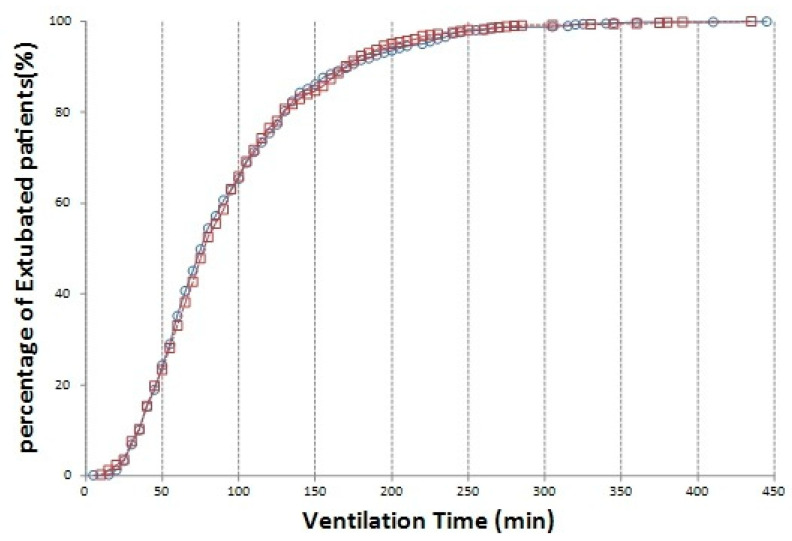
Comparison of ventilation time between on- (blue circles) and off-pump (red squares) group.

The duration of mechanical ventilation was not significantly different between the groups (Figure 3).

There were no significant differences between groups in terms of PACU, IMC or hospital length of stay (Table 2). Furthermore, there was no significant difference in the primary and secondary fast-track failure in the on- compared to off-pump CABG (Table 3). Postoperative complications were also comparable, except for statistically significant differences in in-PACU postoperative bleeding, which was higher for the off-pump group and a higher perioperative and in-PACU red blood cell (RBC) transfusion rate for the on-pump group (Table 3).

## 4. Discussion

In our study, we could find no significant difference between on- vs. off-pump CABG patients managed with a fast-track protocol in a specialized PACU, in terms of time to tracheal extubation, primary and secondary fast-track failure and PACU, IMC, and hospital length of stay. Postoperative outcome and complications were comparable, except for a statistically significant higher blood loss in the off-pump group and higher RBC transfusion rate in the on-pump group.

Most previous studies and meta-analyses compared on- vs. off-pump CABG using conventional perioperative anesthetic management and demonstrated longer postoperative mechanical ventilation time in the on-pump group [1,2]. Only Scott et al. [14] used a fast-track protocol and reported a significantly shorter ventilation time after off-pump surgery (7.4 h vs. 5.8 h). In our study, the extubation time was overall much shorter than that reported by Scott et al. without significant differences between the groups (on-pump 75 min vs. off-pump 80 min). The shorter ventilation time in our study is probably attributable to our fast-track protocol. Additionally, our patients were managed in a specialized PACU with limited opening hours, while in the study by Scott et al., the patients were recovered in an ICU. Probst et al. [16] suggested that the fast-track protocol in specialized PACU leads to shorter mechanical ventilation time when compared to patients managed with the same protocol in ICU, while Graß et al. [17] reported that limited opening hours also resulted in a significantly reduced mechanical ventilation time. Furthermore, Scott et al. included unselected patients undergoing primary CABG. In contrast, we preselected patients according to our fast-track protocol (temperature >36 °C, hemodynamically stable, minimal inotropic support, no signs of bleeding at the end of the operation, agreement of anesthesiologist and surgeon for fast-track protocol) [9].

In our study, the fast-track failure was comparable between the two groups. Fast-track failure rates vary in the literature between 11 and 16% [7] with a peak of 45.5% reported by Kogan et al. [18] due to a variety of definitions and different patient populations. The most common definitions are: mechanical ventilation time more than six hours and/or stay in the ICU >24 h, which mostly matches the definition of primary fast-track failure in this study (Figure 1). Our primary fast-track failure rate was 6% for off-pump and 8.2% for on-pump patients and therefore lower than in previously published studies [7,18]. This might be due to the preselection of patients in our fast-track protocol and due to a different study population. However, there are no studies to date, to the best of our knowledge, comparing fast-track failure rates between on- vs. off-pump CABG.

Interestingly, there was no significant difference between on- and off-pump CABG in terms of PACU, IMC, and in-hospital length of stay. In contrast to our data, previous studies [2,18,19] and systematic reviews [1,3] reported a shorter hospital length of stay after off-pump CABG with conventional perioperative anesthetic management. Similarly, in a fast-track setting, Scott et al. [14] reported a shorter hospital length of stay after off-pump CABG. However, it must be taken into account that different reimbursement strategies, which vary from country to country, may affect hospital length of stay.

In agreement with previously published data, postoperative outcomes and complications were not significantly different in our study population [14,20].

Patients after on-pump CABG had a statistically significant lower in-PACU blood loss compared to patients after off-pump CABG; however, we considered the statistically significant difference of less than 100 mL as not clinically relevant. Our data only represent the blood loss until discharge from PACU and cannot account for the total postoperative blood loss. This is in line with Potger et al. [21] who did not report significant differences in chest drainage during the first 12 h after on- vs. off-pump CABG. Contrary to these findings, patients after on-pump CABG received significantly more RBC transfusion compared to those in the off-pump CABG. This is in agreement with systematic reviews [3,22] which reported a reduced postoperative transfusion rate in off-pump CABG surgery. Shaefi et al. [3] suggested that this might be partly due to hemodilution and hemolysis during CPB. Potger et al. [21] founded that intraoperative hemodilution, but not postoperative bleeding or re-operations for bleeding, is an independent risk factor for RBC transfusion, thus explaining our discrepancies between lower blood loss and more RBC transfusion in the on-pump group.

Our study has several limitations: Due to its retrospective design, we cannot fully exclude the risk of unknown biases. Since this was a single-center study with long-term experience in a specialized fast-track protocol, the data may not apply to other centers using standard perioperative anesthetic management. Furthermore, we included, as mentioned, due to our fast-track protocol, only preselected patients operated upon weekdays between 08:00 a.m. and 07:00 p.m. Finally, handwritten anesthesia and PACU observation charts may give room for misinterpretation and lack of data. To overcome some of these limitations, we included a large population size and performed propensity score matching.

## 5. Conclusions

For CABG patients, the surgical approach does not seem to result in significant differences regarding ventilation times, length of stay in PACU, IMC, or hospital, and fast-track failure when the patients are managed with a specialized fast-track protocol. RBC transfusion was higher in on-pump CABG.

## Figures and Tables

**Table 1:** Baseline characteristics and operative data for patients included in the study. Values are the mean (SD) or number (%).

	On-Pump Group *n* = 926	Off-Pump Group *n* = 926	*p*-Value
Age: years	68.7 (9.6)	68.4 (8.7)	0.494
Gender: female	171 (18.4%)	169 (18.2%)	0.952
BMI	28.8 (4.6)	28.5 (4.4)	0.299
Smoking	420 (45.3%)	399 (43%)	0.349
Logistic EuroSCORE	4.4 (4.4)	4.4 (4.9)	0.995
LV ejection fraction; %	54.4 (10.3)	54.5 (10.3)	0.832
Myocardial infarction	322 (34.7%)	313 (33.8%)	0.695
Cerebrovascular accident	56 (6%)	59 (6.3%)	0.847
COPD	40 (4.3%)	34 (3.6%)	0.553
Diabetes mellitus	386 (41.6%)	373 (40.2%)	0.570
Pulmonary hypertension	149 (16%)	182 (19.6%)	0.052
Arterial hypertension	892 (96.3%)	887 (95.7%)	0.632
Peripheral vascular disease	182 (19.6%)	183 (19.7%)	>0.999
NYHA			
NHYA I	162 (17.4%)	154 (16.6%)	0.665
NYHA II	411 (44.3%)	428 (46.2%)	0.455
NNYHA III	331 (35.7%)	325 (35.1%)	0.808
NHYA IV	22 (2.3%)	19 (2.0%)	0.752
Preoperative creatinine; µmol/L	93.7 (50)	91 (38)	0.221
Duration of surgery; min	208.2 (61)	209.2 (60)	0.276

BMI = body mass index; ASA =American Society of Anesthesiologists physical status classification system; LV = left ventricle; COPD = chronic obstructive pulmonary disease; NYHA = New York Heart Association Functional Classification.

**Table 2:** Postoperative outcome parameters for patients included in the study. Values are median (IQR (range)).

	On-Pump Group *n* = 926	Off-Pump Group *n* = 926	*p*-Value
Ventilation time, min	75 (55–120 (65))	80 (55–120 (65))	0.973
PACU LOS, min	260 (255–315 (105))	255 (210–310 (100))	0.702
Intermediate care unit LOS, h	28.5 (17–65 (47))	32.8 (18–67 (49))	0.237
Hospital LOS, d	8 (7–11 (4))	8 (7–11 (4))	0.069

LOS—length of stay; PACU—postanesthetic care unit.

**Table 3:** Postoperative outcome and complications for patients included in the study. Values are the mean (SD) or number (proportion).

	On-Pump Group *n* = 926	Off-Pump Group *n* = 926	*p*-Value
Primary FTF	76 (8.2%)	56 (6%)	0.071
FTF and re-do due to bleeding	9 (1%)	4 (0.4%)	0.178
Secondary FTF	110 (12.9%)	107 (12.3%)	0.702
Re-intubation	9 (1.0%)	7 (0.7%)	0.610
Lactate, µmol/L	1.4 (1.0)	1.4 (0.9)	0.261
In-PACU postoperative bleeding, ml	234 (243)	323 (247)	<0.001
Number of patients having received RBCs transfusion (perioperative)	333 (35%)	208 (22%)	<0.001
Total of transfused RBC units (perioperative)	930	564	
Perioperative RBCs transfusion, units/patient	1 (1.7)	0.6 (1.4)	<0.001
Number of patients received RBCs transfusion (PACU)	100 (11%)	50(5%)	<0.001
Total of transfused RBC units (PACU)	147	82	
In-PACU RBCs transfusion, units/patient	0.15 (0.8)	0.1 (0.4)	0.024
Highest serum creatinine, µmol/L	89.7 (117)	89.1 (110)	0.897
Postoperative renal dialysis	27 (2.9%)	16 (1.7%)	0.092
Cardiac arrhythmias	209 (22.5%)	193 (20.8%)	0.367
Cerebrovascular accident	12 (1.3%)	10 (1%)	0.675
Myocardial infarction	1 (0.1%)	5 (0.53%)	0.124
Mortality	18 (1.9%)	22 (2.3%)	0.528

FTF = fast-track failure; RBCs = red blood cells.

## Data Availability

The datasets generated and analyzed during the current study are available from the corresponding author on reasonable request.
